# Simulation of Internal Defects in TKX-50 Crystals

**DOI:** 10.3390/ma16114063

**Published:** 2023-05-30

**Authors:** Siqi Qiu, Xue Zhao, Yuanyuan Li, Wenyuan Ding, Junrui Huang

**Affiliations:** State Key Laboratory of Explosion Science and Technology, Beijing Institute of Technology, Beijing 100081, China; 3220200108@bit.edu.cn (S.Q.); 3120200209@bit.edu.cn (Y.L.); 3220210106@bit.edu.cn (W.D.); 3220220109@bit.edu.cn (J.H.)

**Keywords:** TKX-50, crystal defects, molecular dynamics, sensitivity, mechanical property

## Abstract

1,1′-Dihydroxy-5,5′-bi-tetrazolium dihydroxylamine salt (TKX-50) is a new type of high-energy low-sense explosive with great application value, but TKX-50 made directly from the reaction has problems such as irregular crystal morphology and relatively large length-diameter, and these factors seriously affect the sensitivity of TKX-50 and limit its large-scale application. The internal defects of TKX-50 crystals have a great influence on their weakness, and studying its related properties is of great theoretical significance and application value. To further investigate the microscopic properties of TKX-50 crystals and to explore the connection between microscopic parameters and macroscopic susceptibility, this paper reports the use of molecular dynamics simulations to construct TKX-50 crystal scaling models with three types of defects—vacancy, dislocation and doping—and conducts molecular dynamics simulations. The influence of TKX-50 crystal defects on the initiation bond length, density, bonding diatomic interaction energy, and cohesive energy density of the crystal was obtained. The simulation results show that the models with a higher bond length of the initiator bond and higher percentage activated the initiator’s N-N bond and lowered the bond-linked diatomic energy, cohesive energy density, and density corresponding to higher crystal sensitivities. This led to a preliminary connection between TKX-50 microscopic model parameters and macroscopic susceptibility. The results of the study can provide a reference for the design of subsequent experiments, and the research method can be extended to the research work on other energy-containing materials.

## 1. Introduction

TKX-50 is a new type of high-energy explosive with high nitrogen content, positive enthalpy of production and high density, green and properties, low toxicity to humans, ease of synthesis, and good thermal stability and low mechanical susceptibility, which has potential applications in the field of high-energy explosives and solid propellants [1,2,3,4]. However, the crystal growth process may be affected by disturbance from external factors, which leads to the presence of defects in the explosive crystals [5,6,7]. The presence of crystal defects can affect the performance of explosives, such as stability, susceptibility and energy characteristics, which further affects the safety and operational performance of weapons and munitions [8,9]. Therefore, studying the effects of crystal defects on the performance of explosives is of great theoretical and practical importance.

With the rapid development of technology and the continuous improvement in computer performance, the rapid development of computing power, and the reduction in computing resource prices, more and more researchers are using the methods of computational materials science to simulate the molecular size of explosives [10,11]. Compared with experiments, the method of simulating explosive crystal morphology using computers has advantages such as environmental protection, low cost, and safety [12,13]. Although this simulation method has certain limitations and cannot completely replace experiments, unlike experimental methods, computer simulation can observe the behavior details of solvents and solutes in the microscopic field [14,15]. Computer simulation methods can provide approximate prediction results before the experiment and can simulate various phenomena that occur throughout the entire experimental process after the experiment, further explaining the experimental phenomena at the micro level, and providing powerful supplements and important references for the experimental results [16,17].

The well-known computational atomic simulation techniques include quantum mechanics (QM) calculations [18], classical Monte Carlo (MC) [19], classical molecular dynamics (MD) [20], and ab initio quantum mechanics methods, which provide reliable predictions [21]. Compared with other methods, the molecular dynamics (MD) method has been applied in various disciplines such as chemistry, physics, and biology and can be used to calculate multi-body motion problems such as complex atomic and molecular systems [22,23]. With the rapid development and updating of computer technology, molecular dynamics simulation methods have also been applied in various fields such as polymer material modification, chemical reaction processes, and drug research and development [24]. The main research object of the molecular dynamics simulation method is a multi-particle system composed of atomic nuclei and extranuclear electrons [25,26]. With the help of a computer, the process of atomic nuclei motion is simulated, the force situation of each particle in the system is analyzed, and their position and velocity at a certain moment are solved to determine the motion state of particles [27]. Then, the structure and properties of the system are calculated, and each nucleus is considered to move according to Newton’s law under the action of all other nuclei and electrons [28]. The basic principle of molecular dynamics simulation is to first establish a cubic box containing only a certain number of molecules with periodic boundary conditions and then start from a certain set of location energy models of the system; solve the Newton equation of motion for all molecules in the box through computer calculation, record their force, speed, and position at different times, along with other information data and then calculate the migration properties. Structural properties, thermodynamic properties, and the corresponding macroscopic properties can be obtained from the microscopic properties of particles in the system [29,30]. The application of this method can not only verify the correctness of the theory but also compare the obtained results with experimental values to verify and improve the model. Additionally, it can simulate certain limiting conditions or system situations that cannot be achieved experimentally [31]. A certain amount of solvent may be mixed in the recrystallization preparation of TKX-50 explosives, which may lead to the impurity of the prepared samples, i.e., the presence of adulteration defects in the explosives [32]. In addition, the crystal growth process may be affected by the interference of various factors, which leads to the presence of vacancy defects and dislocation defects in the explosive crystals [33,34,35]. Jing Li et al. [6] studied the thermal decomposition and oxidation processes of normal TKX-50 (N-TKX-50) and twin-defective TKX-50 (T-TKX-50) using a molecular dynamics simulation. The thermal decomposition and oxidation mechanisms of N-TKX-50 and T-TKX-50 were analyzed in terms of potential energy, reaction pathways, intermediate products and final products. The results showed that the decomposition and consumption rates of T-TKX-50 were higher than those of NTKX-50 at the same temperature, while the decomposition and oxidation end products of N-TKX-50 and T-TKX-50 were the same, which confirmed that the twin crystal defects were detrimental to the thermal stability of TKX-50. Previous studies have mainly focused on the effect of crystal defects on one aspect of the performance of explosives, but relatively few reports have studied these effects comprehensively.

To study the effect of crystal defects on the performance of TKX-50 explosives, this article details the use of Materials Studio 8.0 software to develop models of explosives without and with crystal defects (vacancies, dislocations, and doping); molecular dynamics methods [36,37,38] were then used to predict and compare the bond lengths, densities, bond-linked diatomic interaction energies and cohesion energy densities of the initiating bonds of various models. In addition, the effect of crystal defects on the performance of explosives was studied. A comprehensive evaluation of the effect of crystal defects on the performance of explosives was carried out. The results of the study can provide theoretical guidance for the performance evaluation of explosives.

## 2. Calculation Models and Methods

### 2.1. Establishment of the Initial Model of TKX-50 Crystal

**Figure 1:**
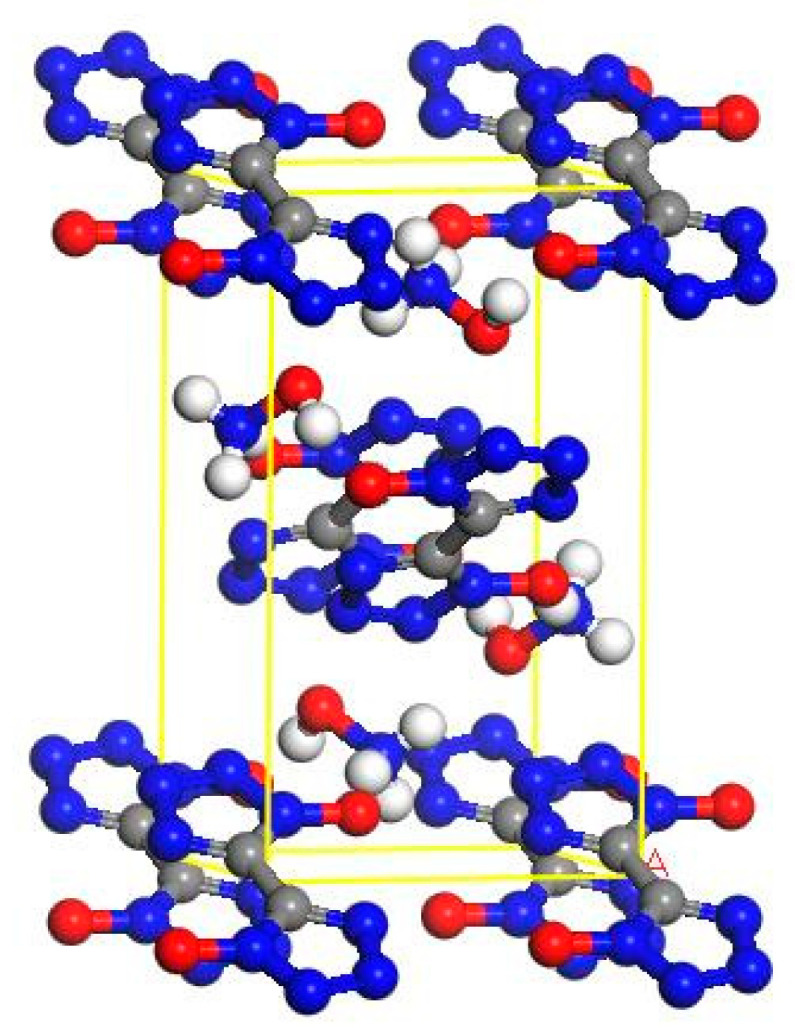
TKX-50 single-crystal-cell model.

**Figure 2:**
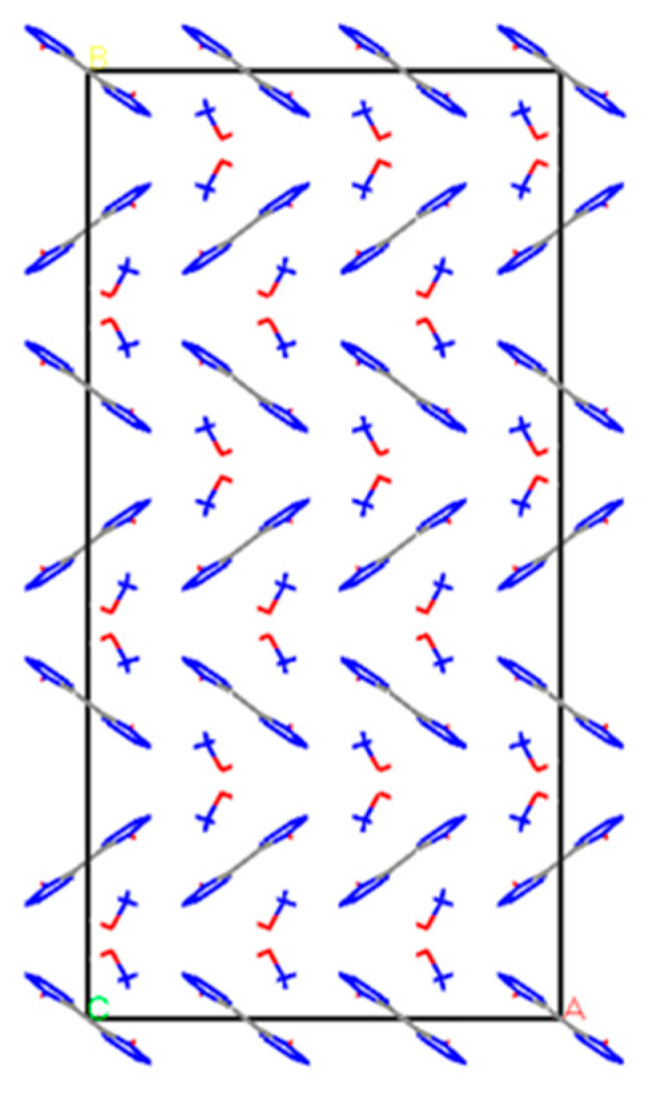
TKX-50 explosive supercell model (Model 1).

TKX-50 explosive belongs to a monoclinic crystal system with the space group P21/c and lattice parameters of a = 5.4408 Å; b = 11.7514 Å; c = 6.5612 Å; α = γ = 90°; β = 95.071° [39,40]. TKX-50, an energy-containing ionic salt, contains in a single-cell two-1,1′-di hydroxy-5,5′-bistetrazole anion and four hydroxylamine cations with an overall electrically neutral nature [41,42]. An individual cell model of the TKX-50 explosive is shown in Figure 1. The TKX-50 single-cell model was extended to a (3 × 3 × 3) supercell model containing 54 1,1′-dihydroxy-5,5′-bistetrazole anions with 108 hydroxylamine cations, for a total of 162 ions. This is shown in Figure 2. To facilitate comparison with the model containing crystal defects, the crystal model without crystal defects is labeled as Model 1.

### 2.2. Modeling of TKX-50 Crystal Defects

The crystal defects in this study include a total of three types, namely vacancies, dislocations and doping.

**Figure 3:**
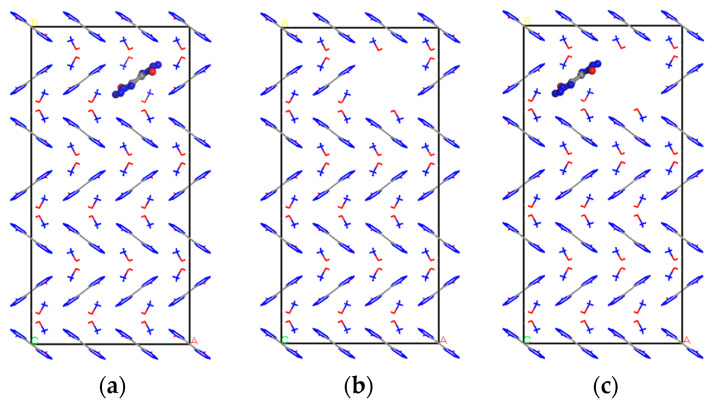
Cell model of TKX-50 with vacancy defects. (**a**) Model 2, (**b**) Model 3, (**c**) Model 4.

By deleting two 1,1′-dihydroxy-5,5′-buntetrazolium anions and four hydroxylamine cations from the second layer of the defect-free supercell model, a total of six ions were obtained, resulting in a defective crystal model containing 3.70% vacancies, as shown in Figure 3a, labeled as Model-2.

Similarly, three 1,1′-dihydroxy-5,5′-bietetrazole anions and six hydroxylamine cations were deleted, respectively, for a total of nine ions, to obtain a defective crystal model containing 5.56% vacancies, as shown in Figure 3b, labeled as Model 3. Four 1,1′-dihydroxy-5,5′-Biotetrazole anions with eight hydroxylamine cations, for a total of twelve ions, were used to obtain a defective crystal model containing 7.41% vacancies, as shown in Figure 3c, labeled as Model 4.

**Figure 4:**
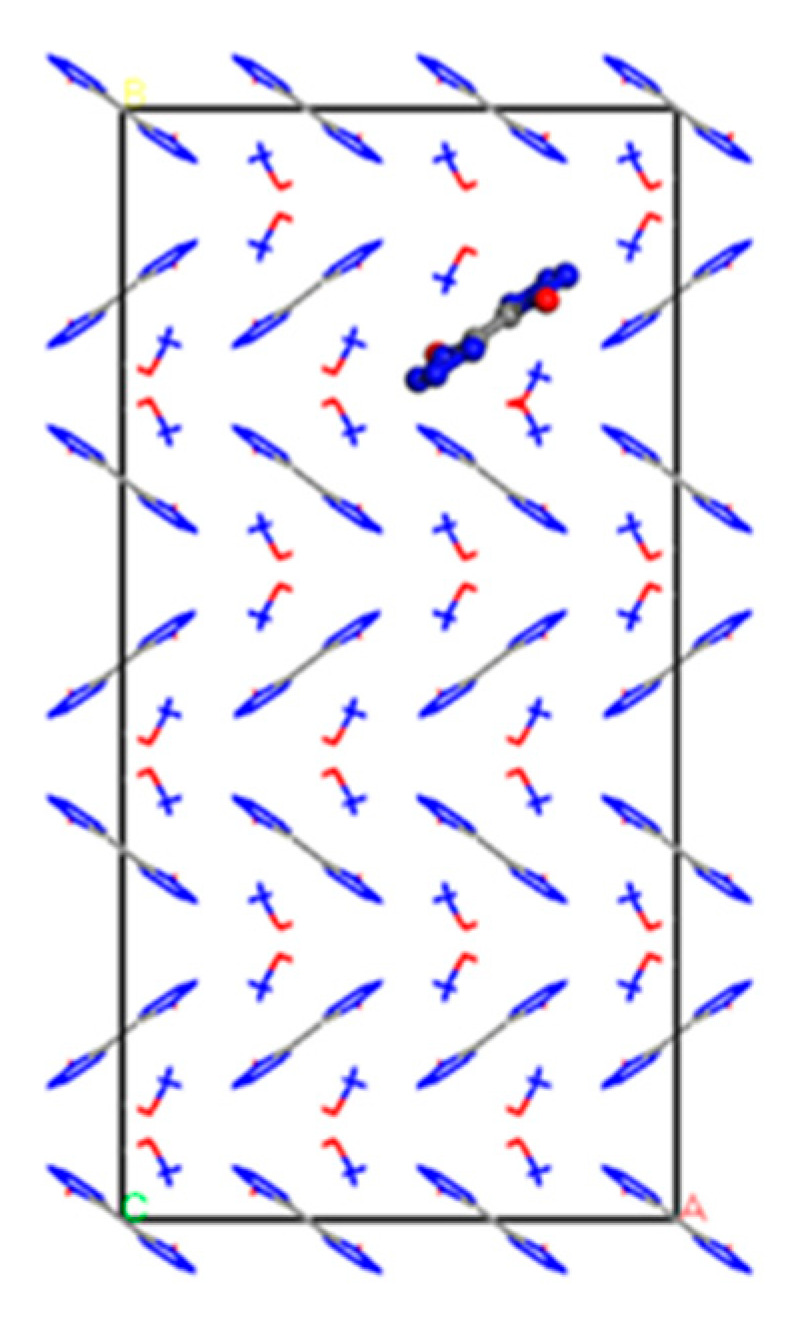
Cell model of TKX-50 with dislocation defects (Model 5).

The three 1,1′-dihydroxy-5,5′-bistetrazole anions and six hydroxylamine cations in the second layer of the defect-free crystal model were shifted downward by a distance of 1 Å to obtain a crystal model containing dislocation defects with a defect rate of 5.56%, as shown in Figure 4 and labeled as Model 5.

**Figure 5:**
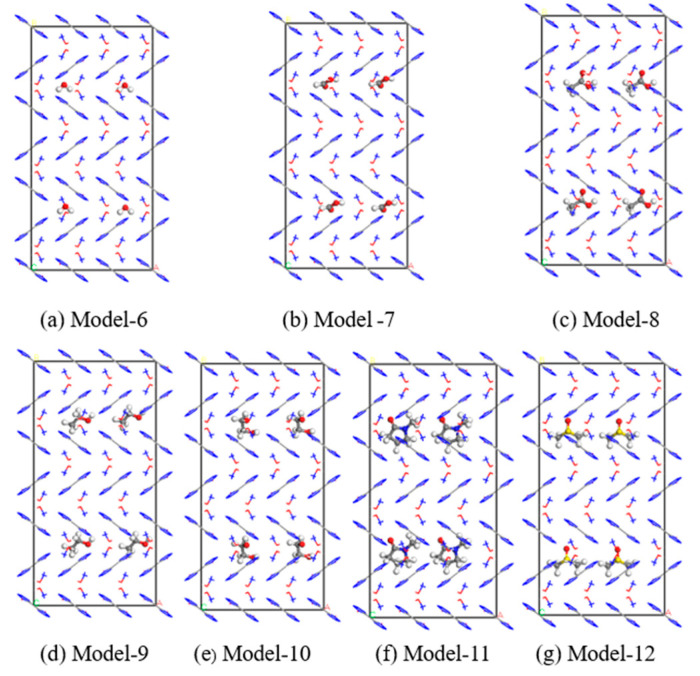
Cell model of TKX-50 with doping defects.

Four water molecules were inserted into the supercell model to obtain a defective crystal model containing doping defects, as shown in Figure 5a, labeled as Model 6.

Similarly, four formic acid molecules, acetic acid molecules, ethanol molecules, ethylene glycol molecules, N-methyl-pyrrolidone (NMP) molecules and dimethyl sulfoxide (DMSO) molecules were inserted into the supercell model, as shown in Figure 5b–g, labeled as Model 7, Model 8, Model 9, Model 10, Model 11, and Model 12, respectively.

### 2.3. Calculation Conditions

The initial crystal model of TKX-50 explosives and the crystal model containing defects were subjected to energy minimization to eliminate internal stresses, while molecular dynamics calculations were performed afterward, where the temperature was set to 298 K, the pressure was set to 0.0001 GPa, and the constant temperature and pressure (NPT) system synthesis with the PCFF force field was chosen [43]. The initial molecular velocity of motion was determined by the Maxwell–Boltzmann distribution and the solutions of the Newtonian equations of motion were based on the basic assumptions of periodic boundary conditions, and time-averaged equivalence to the system synthesis average. The integration was performed using the Verlet method [44]. The temperature was controlled using the Andersen method, the pressure was controlled using the Parrinello method, the van der Waals force (vdW) was calculated using the atom-based method, and the electrostatic interaction was calculated using the Ewald method with a truncation radius of 15.5 Å and a truncation tail correction [45]. The time step was set to 1 fs, and the total number of simulation steps was 200 ps, of which the first 100 ps were used for thermodynamic equilibrium and the last 100 ps for statistical analysis. During the simulation, the trajectories were saved every 500 fs, yielding a total of 400 frames of trajectory files.

## 3. Analysis of Results

### 3.1. Equilibrium Discriminant and Equilibrium Structure

**Figure 6:**
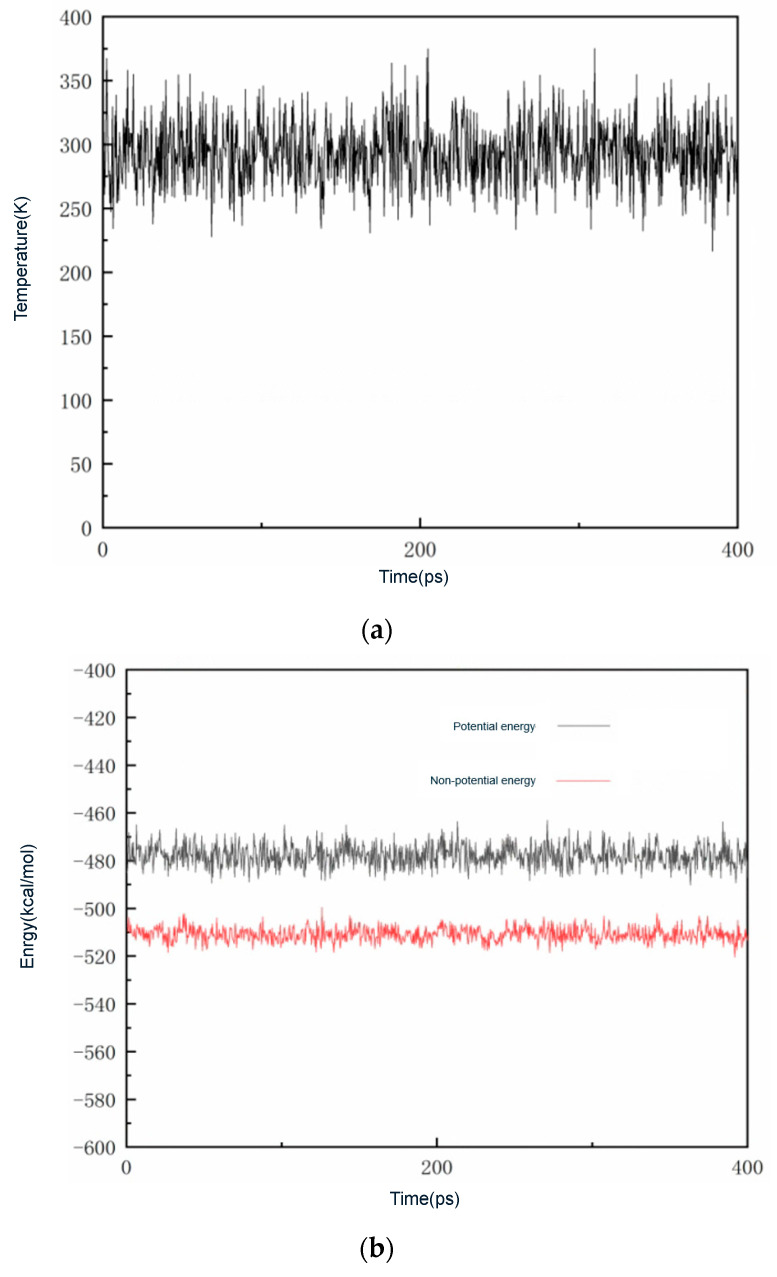
Curve of temperature versus energy with time. (**a**) T–t curves. (**b**) E–t curves.

In extracting the simulation results, the hybrid system needs to be brought into equilibrium, and when the system is in equilibrium, both temperature equilibrium and energy equilibrium must be satisfied. The system is usually considered to have reached equilibrium when the temperature and energy fluctuation range are at 10%. Taking the doping defect model, Model 4, as an example, Figure 6 gives the temperature and energy variation curves of the mixed system with time during the simulation.

As shown in Figure 6, in the process of simulating molecular dynamics, the temperature and energy variation curves with time fluctuate near the equilibrium point and the wave amplitude is not large. At this point, the simulation system can be considered to be in an equilibrium state, and the simulation results have reference values.

### 3.2. Lead Key Build Length

The so-called initiation bond is the lowest energy and weakest chemical bond in energy-containing materials. When subjected to external stimuli, the initiating bonds are most likely to break, leading to the decomposition or explosion of energy-containing materials. The initiating bonds in TKX-50 and its defective crystals are N-N bonds [6], so N-N bonds were chosen to predict the susceptibility of different systems.

**Figure 7:**
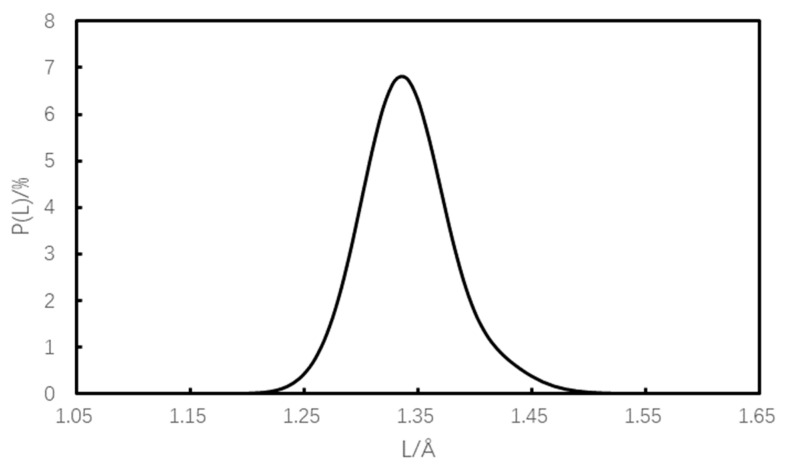
Bond length distribution of the induced bonds (Model 2).

The bond length distribution of the initiating bonds in the equilibrium system after molecular dynamics simulation is given in Figure 7 (the horizontal coordinates indicate the bond lengths of the initiating bonds and the vertical coordinates indicate the probability of bond length distribution), taking the vacancy defect Model 2 as an example. The most available bond lengths (L_prob_), the average bond length (L_ave_), and the maximum bond length (L_max_) corresponding to different models at equilibrium are listed in Table 1.

Figure 7 shows that the bond-length distribution of the initiating bonds (N-N bonds) has an approximately symmetric Gaussian distribution when the mixed system reaches equilibrium. As can be seen from Table 1, when the system reaches equilibrium, for different crystal models, the most variable bond lengths are approximately equal and the average bond lengths vary in a small range, indicating that the effect of crystal defects on the most variable bond lengths and average bond lengths is small, but the range of variation of the maximum bond lengths is obvious. For the defect-free crystal model (Model 1), the maximum bond length of the initiating bond is the smallest, at 1.550 Å, while the maximum bond lengths of the defective crystals are all larger than the corresponding maximum bond-length values of the defect-free crystals. For the different defective crystal models, the vacant defective crystal model (Model 2) has the smallest bond length of 1.572 Å, and the doped defective model (Model 11) has the largest bond length of 2.120 Å. The increase in the maximum bond length compared to the defect-free crystal model ranges from 1.42% to 36.77%. The increase in the maximum bond length indicates that the activity of the initiating bond is enhanced, the sensitivity of the explosive is increased, and the safety is weakened. On this basis, it can be seen that among the different defect models, the N-methylpyrrolidone (NMP) doped defect model (Model 11) has the largest maximum bond length, the highest susceptibility, and the worst safety, which indicates that N-methylpyrrolidone (NMP) doping defects have a more significant impact on the maximum bond length of TKX-50 crystals. It can also be seen that the crystal model with doping defects corresponds to the highest maximum bond length, followed by the dislocation defect model, followed by the vacancy defect model. In addition, it can also be seen that the maximum bond length of the initiating bond gradually increases with the increase in vacancy defects in the explosive, indicating that the susceptibility of the explosive gradually increases, i.e., the safety of the explosive gradually decreases with the increase in crystal defects, predicting that crystal defects will adversely affect the safety of the explosive.

### 3.3. Bonded Diatomic Action Energy

The bond-linked diatomic energy is mainly used to reflect the strength of the bond. The higher the bond-linked diatomic energy, the stronger the bond, the lower the sensitivity of the energy-containing material, and the better the safety [46]. The bond-linked diatomic energy (E_N-N_) is calculated as follows:

$$E_{N-N\;}=\frac{E_{T}-E_{F}}{n}$$

where *E_T_* is the total energy of the system when the explosive reaches the equilibrium state, kcal·mol^−1^; *E_F_* is the total energy of the system after fixing all the N atoms in TKX-50, kcal·mol^−1^ and *n* is the number of N-N bonds contained in TKX-50 in the system.

**Figure 8:**
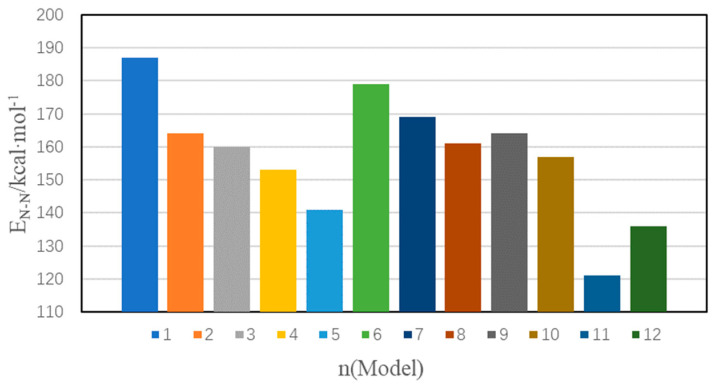
Bond-linked diatomic interaction energies for different crystal models.

Based on the results of molecular dynamics calculations, the bond-linked diatomic interaction energies of different models were obtained, and the results are shown in Figure 8.

As can be seen from Figure 8, among all models, Model 1 has the highest bond diatomic interaction energy at 187.49 kcal·mol^−1^, while Model 11 has the lowest bond diatomic interaction energy at 121.43 kcal·mol^−1^. Compared with Model 1, the reduction in the interaction energy of bonded diatoms is 4.40% to 35.23%. The decrease in the interaction energy of bonding diatoms indicates that the initiating bond is weaker in strength and prone to fracture, which increases the sensitivity of the explosive and reduces its safety. Among all the doping defect models, Model 11 has the smallest bonding diatomic interaction energy, the highest sensitivity, and the worst safety, indicating that NMP doping defects have the most significant impact on the sensitivity of explosives among all the doping defect types. In addition, it can be seen that compared to vacancy defects and dislocation defects, the bonding diatomic interaction energy of dislocation defects is smaller, indicating that dislocation defects are more likely to affect the bonding diatomic interaction energy of TKX-50 crystal.

### 3.4. Internal Cohesive Energy Density

Cohesive energy density (CED) is defined as the work performed to overcome the intermolecular forces when 1 mol of a substance changes from a condensed to a gaseous state in a unit volume. The cohesion energy density is a non-bonding force and is numerically equal to the sum of the van der Waals force (vdW) and the electrostatic force (electrostatic) [47]. The cohesion energy density, van der Waals force and electrostatic force for different models are obtained by molecular dynamics calculations, and the results are shown in Table 2.

As can be seen from Table 2, among the different crystal models, the defect-free crystal model (Model 1) has the largest cohesive energy density, van der Waals force, and electrostatic force are 0.764 kJ·cm^−3^, 0.059 kJ·cm^−3^ and 0.705 kJ·cm^−3^, respectively. Among the defect models, the cohesive energy density of the doped defect model (Model 6) was the largest, at 0.743 kJ·cm^−3^, while the cohesive energy density of the doped defect model (Model 11) was the smallest, at 0.661 kJ·cm^−3^, with the reduction in cohesive energy density ranging from 2.75% to 13.48%. The decrease in cohesive energy density indicates that the explosive’s sensibility increases and its safety decreases. In the doping class defect model, the N-methylpyrrolidone (NMP) doping defect model corresponds to the smallest cohesive energy density, with the highest susceptibility and the worst safety, which also indicates that the N-methylpyrrolidone (NMP) doping defect has a more significant effect on the internal cohesive energy density. In addition, it can also be seen from Table 2 that the dislocation defect has a lower cohesive energy density compared to both the vacancy defect and the dislocation defect, indicating that the dislocation defect is more likely to affect the susceptibility of the explosive. This further shows that when the defects in the explosive increase, the cohesive energy density gradually decreases, which predicts that the explosive’s susceptibility gradually increases.

### 3.5. Density

Density, a common burst parameter, is closely related to the sensitivities of TKX-50 crystals. The lower the density, the higher the susceptibility and the lower the safety performance [48]. Considering the N-N bond as the initiating bond of the TKX-50 crystal, the longer the bond is, the easier it is to initiate the decomposition. Taking the average bond length of each model as the benchmark, the ratio of bond lengths larger than the average bond length to all bond lengths was counted, and a new parameter was introduced as the activation bond percentage R_act_. The density and R_act_ of different models were obtained using molecular dynamics calculation, and the results are shown in Table 3.

From Table 3, it can be seen that among the different crystal models, the defect-free crystal model (Model 1) has the highest density at 2.0590 g·cm^−3^. Among the defect models, the vacancy defect model (Model 2) has the highest density at 1.9828 g·cm^−3^, and the dislocation defect model (Model 5) has the smallest density at 1.8598 g·cm^−3^, and the density reduction ranges from 3.70% to 9.67%. The decrease in density indicates that the explosive’s susceptibility increases and its safety decreases. In the doping class defect model, the N-methylpyrrolidone (NMP) doping defect model corresponds to the smallest density with the highest susceptibility and the worst safety, which also indicates that the N-methylpyrrolidone (NMP) doping defect has a more significant effect on the susceptibility of explosives. In addition, it can also be seen from Table 3 that as the crystal defect rate rises, the percentage of activated N-N bonds larger than the average bond length also rises, which indicates that the number of N-N bonds that can easily be triggered to detonate increases and the crystal susceptibility increases. This is also consistent with the actual situation from the data, but it is more difficult to experimentally verify because it is more difficult to measure the N-N bond length distribution in crystals. Based on the available simulations, it is clear that TKX-50 crystal susceptibility increases as the defect rate increases, and the higher the defect rate the lower the crystal density and the higher the crystal susceptibility.

## 4. Conclusions

This article used molecular dynamics methods to construct a scaled model of TKX-50 crystals without defects and containing internal defects and conducted relevant simulations to obtain the influence of TKX-50 crystal defects on the bond length, density, bond bonding energy, and cohesive energy density of the crystal. The conclusion is as follows.

1. The minimum bond length of defect-free crystals is 1.550 Å, the maximum bond-linked diatomic energy is 187.49 kcal·mol^−1^, and the maximum cohesive energy density is 0.764 kJ·cm^−3^; the bond length of defective crystals increases compared with that of defect-free crystals, with an increase of 1.42~36.77%; the bond-linked diatomic energy and cohesive energy density decrease, with decreases of 4.40~35.23% and 2.75~13.48%, respectively. The decrease in bonding diatomic interaction energy and cohesion energy density is 4.40~35.23% and 2.75~13.48%, respectively. This shows that with an increase in initiation bond length and a decrease in bond-linked diatomic energy and cohesive energy density, the sensitivity of the explosive increases and the safety decreases.
2. The density of TKX-50 crystal without defect-type crystal is a maximum of 2.0590 g·cm^−3^, the activation bond ratio (R_act_) is a minimum of 14.56%, and the density of defective crystal gradually decreases and shrinks by 3.70–9.67%; as the defect rate of crystal rises, the activation bond ratio (R_act_) of the N-N bond, which is larger than the average bond length, also rises, and the crystal density decreases, which indicates that the density of TKX-50 crystals is related to the defect rate, and the larger the defect rate, the lower the density, indicating the increased sensitivity and weakened safety of the explosive.
3. In the defect model without solvent doping, vacancy defects are more likely to affect the explosive susceptibility compared with dislocation defects, thus reducing the safety of explosives; among the seven solvent-doped defect models, N-methylpyrrolidone (NMP) doped defects have the largest bond length of 2.120 Å, the smallest bond-linked diatomic action energy, and cohesion energy density and density, which are 121 kcal·mol^−1^, 0.630 kJ·cm^−3^, and 1.8688 g·cm^−3^, so it has the most significant effect on the explosive sensitivities and is the most likely to reduce the safety of explosives.

This study constructed the correlation between macroscopic crystal sensitivity and microscopic molecular motion through numerical simulation. The research results indicate that molecular dynamics simulation methods can be a powerful tool for exploring the microscopic properties of energetic material crystals. This method can also be extended to research on other energetic materials, providing a reference basis for the optimization experiments of energetic materials.

## Figures and Tables

**Table 1:** Bond lengths of initiating bonds in different crystal models.

Model	L_prob_/Å	L_ave_/Å	L_max_/Å
1	1.399	1.200	1.550
2	1.399	1.205	1.572
3	1.399	1.215	1.574
4	1.340	1.210	1.578
5	1.340	1.215	1.583
6	1.341	1.205	1.560
7	1.340	1.210	1.570
8	1.341	1.240	1.650
9	1.341	1.241	1.690
10	1.340	1.240	1.660
11	1.342	1.245	2.120

**Table 2:** Cohesive energy density, van der Waals force and electrostatic force for different crystal models.

Model	CED/kJ·cm^−3^	vdW/kJ·cm^−3^	Electrostatic/kJ·cm^−3^
1	0.764	0.059	0.705
2	0.738	0.055	0.683
3	0.736	0.054	0.682
4	0.733	0.052	0.681
5	0.683	0.045	0.638
6	0.743	0.055	0.688
7	0.729	0.056	0.673
8	0.718	0.055	0.663
9	0.714	0.054	0.660
10	0.711	0.053	0.658
11	0.661	0.031	0.630

**Table 3:** Density vs. R_act_ for different crystal models.

Model	ρ/g·cm^−3^	R_act_/%
1	2.0590	14.56
2	1.9828	17.01
3	1.9446	20.72
4	1.9065	24.32
5	1.8598	29.49
6	1.9537	16.95
7	1.9410	20.13
8	1.9177	23.42
9	1.8960	25.02
10	1.9148	23.49
11	1.8688	15.74
12	1.8835	26.17

## Data Availability

The data that supports the findings of this study are available within the article.
